# Barriers and Enablers to a Hospital-to-Home, Combined Exercise and Nutrition, Self-Managed Program for Pre-Frail and Frail Hospitalised Older Adults

**DOI:** 10.3390/healthcare12060678

**Published:** 2024-03-18

**Authors:** Chad Yixian Han, Georgia Middleton, Jersyn Doh, Alison Yaxley, Yogesh Sharma, Claire Baldwin, Michelle Miller

**Affiliations:** 1 Caring Futures Institute, College of Nursing and Health Science, Flinders University, Adelaide, SA 5042, Australia; georgia.middleton@flinders.edu.au (G.M.); 22431232@students.latrobe.edu.au (J.D.); alison.yaxley@flinders.edu.au (A.Y.); claire.baldwin@flinders.edu.au (C.B.); michelle.miller@flinders.edu.au (M.M.); 2 College of Medicine and Public Health, Flinders University, Adelaide, SA 5042, Australia; yogesh.sharma@flinders.edu.au; 3 Department of General Medicine, Flinders Medical Centre, Adelaide, SA 5042, Australia

**Keywords:** barriers and enablers, nutrition, exercise, frailty, older adults

## Abstract

Introduction: Self-managed exercise and nutrition interventions can alleviate pre-frailty and frailty but understanding of adherence to them is lacking. This study aimed to explore the experiences of, and barriers and enablers to, a hospital-to-home self-managed combined exercise and nutrition program for hospitalised older adults living with pre-frailty and frailty. Methods: A hybrid approach to data- and theory-driven descriptive thematic analysis identified experiences, barriers, and enablers to participation in a 3-month, self-managed, exercise–nutrition, hospital-to-home frailty-support program. Pre-frail and frail older adult patients ≥ 65 years admitted to the acute medical unit at a South Australian tertiary hospital were recruited. Individual semi-structured interviews were audio-recorded, transcribed verbatim, and analysed descriptively, using the Theoretical Domains Framework. Results: The nutrition component of the program found 11 common barriers and 18 common enablers. The exercise component included 14 barriers and 24 enablers. Intentions, Social influences, Environmental context/resource and Emotions served as primary barriers towards adherence to both components. Common enablers for both components included Knowledge, Social identity, Environmental context/resource, Social influences, and Emotions. Conclusions: This research revealed important factors affecting adherence to a self-managed exercise–nutrition program in pre-frail and frail older adults within the environment, resources, and emotion domains that should be considered when designing other intervention programs in this population group.

## 1. Introduction

Frailty can be defined as a clinical syndrome where three or more of the following conditions are observed: a slow walking speed, unintentional weight loss, exhaustion, or low grip strength and physical activity; pre-frailty is a state prior to frailty where one or more of these symptoms are present [1]. Pre-frailty and frailty are conditions that contribute to late-life disability and loss of independence [2]. Frailty is associated with higher healthcare costs among community-dwelling older adults [3]. This relationship follows a dose–response-increase pattern, with higher costs as the severity of frailty increases [3]. Early exercise and nutrition interventions can alleviate or reverse pre-frailty and frailty, and support community-dwelling older adults to preserve their autonomy at home [4]. A previous review of 11 studies has also demonstrated that such interventions can bring about significant improvements in pre-frailty and frailty and frailty-related health outcomes in older adults that were hospitalised, in particular reducing frailty and some frailty-related physical indicators [5]. For example, the INDEPENDENCE program was an individualised hospital to home, exercise–nutrition self-managed intervention for pre-frail and frail hospitalised older adults [6]. Program participants received an individualised exercise–nutrition care plan while admitted, and that continued for three months post-discharge through an ambulatory self-management service in the form of four home visits and four telephone calls. The intervention demonstrated a 18% improvement in frailty compared to those in standard care, and adherence to the inpatient and home visits/telehealth intervention was high (91%).

Adherence is a key factor in the success of such programs and positive changes are usually associated with level of adherence, i.e., higher adherence with better efficacy. Adherence refers to the extent to which individuals follow a prescribed intervention, and the World Health Organisation (WHO) defines treatment adherence as the extent of an individual’s behaviour, i.e., taking medications and following exercise and diet changes, that is consistent with what was agreed and recommended by the healthcare provider [7]. When individuals do not adhere to treatment interventions, they may miss out on the benefits that they provide. In the case of exercise–nutrition programs for frailty, lower adherence may result in a smaller magnitude of improvement in frailty and frailty-related health outcomes [8]. Adherence to prescribed exercise and nutrition advice can be notoriously low, especially in older adult populations [9]. Therefore, it is important for healthcare providers to identify and address barriers and enablers to adherence when designing and implementing exercise–nutrition interventions for pre-frailty and frailty in older adults.

In brief, barriers are factors that prevent the adoption of said behavioural changes, whereas enablers facilitate them. Connecting barriers and enablers to intervention strategies can help in their implementation and that can be done by the application of a relevant theoretical framework. Moreover, a theoretical framework to identify barriers and enablers affecting health behaviours might be more successful at changing them, when compared to a non-theory-driven one [10]. One of the most used frameworks is the Theoretical Domains Framework (TDF) [11]. The TDF comprises 14 domains from numerous unique theoretical constructs, anchored by theories of behaviours and its changes. These domains can be applied to barriers or enablers of health behaviours, and they include (1) Knowledge, (2) Skills, (3) Social/Professional role and identity (4), Beliefs about capabilities, (5) Optimism, (6) Beliefs about consequences, (7) Reinforcement, (8) Intentions (9) Goals, (10) Memory, attention, and decision processes, (11) Environmental context and resources, (12) Social influences, (13) Emotion, and (14) Behavioural regulation. The TDF has also been used in the context of frailty [12]. A recent qualitative meta-synthesis of ten nutrition interventions (including six that were nutrition–exercise) for older adults with physical frailty found that themes emerged at the individual level (knowledge, behaviour, physical changes), external-environment level (social support, physical and human environment, socioeconomic), and intervention-related level (intervention protocol, implementation personnel, and products) [13].

Identifying barriers and enablers to treatment adherence can ensure that the design of future interventions can be as effective as they are efficacious, by reducing the lack of effect caused by poor adherence. Understanding barriers and enablers can serve as a valuable guide for the development and implementation of health services, particularly those of transitional care nature, from hospital to home. The objective of this study was to address the above mentioned point, by exploring the experiences of, and barriers and enablers to, a self-managed combined exercise and nutrition program from the point of view of hospitalised older adults living with pre-frailty and frailty. This is one of the first qualitative reports from hospitalised older adults who took part in such a hospital-to-home self-management program.

## 2. Materials and Methods

### 2.1. Design

#### 2.1.1. Theoretical Framework

In accordance with recommendations for qualitative inquiry, data were collected and analysed simultaneously [14]. This allowed the research team to solidify the findings through identifying directions and gaps. A hybrid thematic approach consisting of both data- and theory-driven concepts and analyses was used. As such, the TDF was drawn on to distinguish behavioural elements associated with barriers and enablers influencing adherence to the exercise and nutrition components of the program [11].

#### 2.1.2. Demonstrating Trustworthiness and Rigour

The qualitative methods and findings in this study are reported in accordance with the COREQ (COnsolidated criteria for REporting Qualitative research) statement/checklist (see Supplementary File S1) [15]. Interview questions and prompts were developed with the guidance from the research team from multidisciplinary backgrounds—physiotherapy, nutrition and dietetics, and medicine, and with expertise in the management of pre-frail and frail older adults. The interviewer had experience of delivering nutrition–exercise intervention to pre-frail and frail older adults, and prior training and experience in conducting semi-structured interviews [16]. The research team and reflexivity, i.e., personal characteristics and relationships with participants, are important aspects in demonstrating the trustworthiness and rigour of a qualitative study [15]. All authors have experience in qualitative research methods, with backgrounds in physiotherapy, nutrition and dietetics, medicine, or psychology. All authors had a thorough understanding of data collection and those directly involved with participants were competent in the clinical management of pre-frail/frail older adults within their professional scope. This fostered a trusting relationship between the experiment facilitators and patients the in order to produce accurate findings and preserve the quality of the research. Of the seven authors, five were female and two were male.

#### 2.1.3. Design of Interview Protocol

To explore the barriers and enablers of pre-frail and frail hospitalised older adults to a hospital-to-home, combined exercise and nutrition, self-managed program, a semi-structured, in-depth interview protocol was designed to allow participants to freely express their personal views. Interview questions was developed collectively by the team and addressed the following areas (see Supplementary File S2 for the full interview guide):

- Experience of program—what were their overall feelings and opinions towards the program?
- Barriers and enablers to diet—what helped or deterred them from adhering to the diet recommendations/changes?
- Barriers and enablers to exercise—what helped or deterred them from adhering to the exercises prescribed?

### 2.2. Settings and Participants

Purposive sampling was employed to recruit hospitalised older adults that were pre-frail or frail, as determined by the Edmonton Frail Scale (EFS) [17], and that took part in a hospital-to-home, self-managed, combined exercise and nutrition intervention program between September 2020 and June 2021. Purposive sampling was chosen because it can yield the most appropriate information by selecting a population that is most relevant to answer the research questions in this study, i.e., older adults interviewed were the most appropriate to discuss their experiences of barriers and enablers to the program, as they were participants [18,19].

Inclusion criteria for selection into the aforementioned self-managed program included: Older adults ≥ 65 years; cared for by the local health network; an EFS score ≥ 6; able to understand English instructions; without cognitive impairment (standardised mini-mental state examination (MMSE) ≥ 25); and had access to a mobile or home phone. Those receiving palliative care, on home oxygen, or assessed by their treating physician as not safe to participate were excluded.

### 2.3. Procedure

Participants’ demographic information was collected through medical records provided by Flinders Medical Centre in Adelaide, as part of the baseline data collection from the pilot randomised controlled trial. All interviews were conducted in the homes of the participants to ensure their privacy and to allow them to freely express their personal views. Participants were also interviewed privately, in the absence of other family members, where pseudonyms were used to ensure anonymity in the recording. The interviews were audio recorded and transcribed verbatim, using a software program (Microsoft word version 16). Audio recordings were checked for accuracy, and initial analytical notes were made before the formal process of coding was undertaken Participants were recruited regardless of program completion or degree of adherence to ensure an accurate representation of data.

### 2.4. Data Analysis

The first author (C.Y.H.) transcribed the interviews, and the transcriptions were cross-checked by another member of the research team (J.D.). A hybrid approach to descriptive thematic analysis was undertaken, whereby data-driven and theory-driven codes and concepts were generated and used to identify experiences, barriers, and enablers related to the program [20,21]. The transcripts were repeatedly read to gain familiarity with the data.

The first round of coding followed an inductive coding process, where lines of text were labelled with a code that was descriptive of the text meaning. This process allowed us to stay true to the data and ensure an accurate representation of participant experiences. To ensure that no theory-driven concepts were missed in the analysis, following the inductive-coding round, we conducted a round of deductive coding, where transcripts were read and further coded using TDF language and terminology [11]. This involved generating new codes against lines of text that had not been coded, adapting inductive codes to reflect the TDF terminology, or grouping inductive codes within deductively generated TDF codes. The codes were then categorised into ‘experiences’, representing the participant’s overall experience of participating in the INDEPENDENCE program, ‘barriers’, representing what participants described as challenging or difficult for them to participate or adhere to in the program, and ‘enablers’, representing what participants described as facilitating or helping them participate or adhere to the program. Following this, all codes within each category were mapped and categorised within the TDF domains.

This hybrid approach was undertaken to ensure that the data-driven concepts were maintained, while positioning the findings within the theory-driven concepts of the TDF [22]. C.Y.H. was responsible for data analysis, and the coded transcripts were checked by two other members of the research team (C.B., A.Y.). Discussions with the research team were carried out until an agreement was reached for any coding discrepancies.

## 3. Results

### 3.1. Participants

A total of 11 pre-frail and frail older adults aged 68–93 years (mean age, 80.4 years; female 64%; living alone 55%) that participated in the INDEPENDENCE trial program were interviewed (Table 1). A range of degrees of frailty were represented in the interview cohort—2/11 pre-frail; 6/11 mildly frail; 2/11 moderately frail; and 1/11 severely frail, as measured through the EFS. Interviews ranged from 15–60 min. Four out of the sixteen total participants recruited in the study did not participate in this study—three were deceased and one declined. Inductively identified codes could fit within domains of the TDF and no additional unique domains were identified.

### 3.2. Experience of the INDEPENDENCE Program

There were both positive and negative experiences of the INDEPENDENCE trial. Sample quotes of the experiences of the program by domains are presented in Table 2 below.

Participants felt that knowledge was improved as they felt an increased awareness towards exercise and diet in general. While some concerns were raised regarding their perceived lack of ability to participate even if they desired to (due to doubts in their own capabilities), others felt optimistic about the program, citing that they looked forward to benefitting and returning to their baseline diet patterns and physical activity levels. There was a sense of group identity as participants felt like part of a program. Some participants also felt that they were rewarded when they were able to return to some pre-hospitalisation activities, such as playing a musical instrument. Participants also overestimated the time available at home to participate in the program, citing follow-up medical appointments as one of the reasons for a lack of time. However, they appreciated the flexibility of the program that helped fit its components into their schedule. Participants reported positive affects for the home visits, and appreciated them, citing them to be helpful. The social influence brought upon by the individualised support also made the experience personal. Regarding the behavioural regulation, participants stated that habits were formed as they reported to have integrated components of the program into their daily routine.

### 3.3. Barriers to the Self-Managed, Exercise/Nutrition, Hospital-to-Home Program

Sample quotes about the barriers to the INDEPENDENCE program by domains are presented in Table 3 below.

### 3.4. Enablers to the Self-Managed, Exercise/Nutrition, Hospital-to-Home Program

Sample quotes about the enablers to the INDEPENDENCE program by domains are presented in Table 4 below.

The barriers reported were from four domains—Intentions; Environmental context/resource; Social influence; and Emotion. Most codes were related to Environmental context/resource and Emotion. Three of four codes within the Environmental context/resource stemmed from past/present illnesses. It was reported that there were pre-existing conditions that restricted some participants diets, e.g., potassium restriction, and conditions that were debilitating and limited their ability to prepare food. The side effects from medications prescribed for those conditions affected dietary intake or adherence. The fourth code identified the lack of transport to obtain groceries, limiting the types of food, as there was no chance to acquire additional food/ingredients that were forgotten in the planned shopping trip. A lack of enjoyment of food and appetite was reported as a barrier towards dietary adherence. Participants felt that there were reduced feelings of hunger and the pleasure of eating/drinking had made it hard to keep to what was recommended in the program. Two codes from the social influence domains were also identified. While the lack of social support and company (loneliness/eating alone) was a barrier to adhering to diet, pressure from peers was also raised as an issue, as participants felt obligated to eat what was given to them when dining in social settings.

Participants’ descriptions of enablers to the nutrition component of the program aligned with the Knowledge, Skills, Social identity, Optimism, Beliefs about consequences, Reinforcement, Goals, Environmental context/resource, Social influences, and Emotion domains of the TDF. There was a general consensus on the improvement of knowledge and skills. Participants reported improved awareness and knowledge of food and protein in health and felt better equipped in terms of skills needed to adhere to the diet recommendations, i.e., the portioning of food. There were codes surrounding Social identity and influences that encouraged dietary adherence. Participants who were caregivers to others felt a responsibility to eat better. The company of a spouse during mealtimes and social support from family members and friends were also cited as positive influences towards keeping to what was recommended in terms of diet. Participants reported to have trust and confidence in their healthcare providers (home-visit therapists) and to perceive nutrition to be something that can improve their weight/strength/blood glucose. The perceived mental and physiological benefit were also raised as enablers to diet adherence, for example, an increased appetite from adhering to the exercise component of the program, as was the attainment of a goal, i.e., weight gain also facilitated better dietary adherence. In some participants, emotions played a part as they experienced increased enjoyment in the food/supplements recommended. There was a general agreement by more than half of the participants on the usefulness of the education resource booklet as a reference guide. Other resources that facilitated dietary adherence were financial support from the government and meal-delivery services.

The barriers reported were from seven domains—beliefs about capabilities, Intentions, Memory, attention, and decision processes, Environmental context/resource; Social influences; Emotion, and Behavioural regulation. Most of the codes rose from the Environmental context/resource domain. Participants described that a lack of sleep, energy, and time all contributed to poor adherence to the prescribed exercises. Participants also stated pre-existing illnesses and injuries as another barrier. Despite the prescribed exercises being indoors, participants quoted cold/wet weather as something that would deter them from exercising that day. Linked to the lack of time “*the day just never seems long enough*” as above mentioned, participants could prioritise social activities over adhering to the exercises if they happened to coincide. For some, the exercises were also hard to adhere to if they were not integrated as part of the participants’ daily routine. In addition, participants reported memory to be an issue, that there could be a lack of cues and that they simply “forgot” about exercising. The participants’ perceived lack of physical capabilities (such as coordination and balance) and low self-efficacy (an individual’s belief in their own capacity to achieve said goal) discouraged them from performing some or at times all exercises. Intrinsically, for some participants they conveyed both explicitly and through implication that a lack of motivation (individual desire to achieve a goal) was a barrier to exercise. One of the emotions that dissuaded participants from exercise was mental stress from dealing with pre-existing illnesses. For example, the stress of having to deal with persistent pain from a physical ailment. From one participant, their report of fear from a pending diagnosis discouraged them from exercising to the prescribed level. Lastly, depression and anxiety were verbalised as factors that prevented adherence to exercises within the program.

Most codes that were identified as enablers to the nutrition component of the program were from the domain Beliefs about consequences. Participants described their perceptions around the positive effects of exercise to maintain or improve strength/health/independence, and that it could also improve their appetite and help prevent falls. They also felt accountable to the healthcare provider supporting them. Similar to enablers to the nutrition component of the program, there was a general consensus that the improvement of knowledge and skills was an enabler to exercise adherence. Participants reported improved awareness of the importance to not be sedentary. They described that the reduced reliance on gait aids, a perception that the level of difficulty of the home exercises were manageable, and learnt skills from their home-visit therapist all helped facilitate adherence. As the exercises were not unfamiliar, participants perceived that they were competent based on previous experience with similar programs. They were also optimistic about the prescribed exercises and that adhering to them would increase their physical capability. This adherence was further encouraged as many expressed that they experienced meaningful physical and health benefits as they progressed through the program. For example, one participant reflected that they could now get up out of their chair and another felt that it benefitted their blood-sugar control. Participants who integrated the exercise regime as part of their daily routine found that it helped them adhere to the exercise component of the program. While some participants stated poor memory as a barrier, others reported that memory was not required as they did not feel like they needed to remember them due to the education resource booklet. The education resource booklet was also described as a cue to exercise. An enabler related to environment was described by a participant who enjoyed options to exercise in an outdoor facility even though home exercises could be done indoors. There were codes surrounding the social identity that encouraged adherence to the exercises. Participants felt the responsibility as a part of the program, contributing individually to a collective group. Two codes from social identity were identified—responsible parents (perceived to be those that took care of their own health and not having to rely on their children) and a worthy patient (perceived that healthcare providers will take individuals who made efforts toward exercising more seriously). Like with the nutrition component, social support from family/partners and healthcare providers facilitated better adherence to exercises within the program. Furthermore, participants described having both intentions and a goal to “reclaim” their life prior to hospitalisation as an enabler towards exercise adherence. A constant decision to stay active and the presence of intrinsic motivation were identified under the domain Intentions.

## 4. Discussion

This study aimed to explore the experience of, and barriers and enablers to, a hospital-to-home self-managed combined exercise and nutrition program for hospitalised older adults living with pre-frailty and frailty. The findings of this study provided insights into the perceptions and lived experiences of pre-frail and frail older adults after a self-managed hospital-to-home support program. Positive experiences were around those of improved knowledge, skills, formed habits, and the program’s social elements. There was a sense of reward when improvement could be felt in participants’ day-to-day activities as the program progressed. Negative experiences were related to a mismatch in expectations versus reality, particularly around personal capabilities, time, and salient events during participation. Many barriers to adherence identified were modifiable by the individualised support aspect of the program. On the same note, what were perceived as enablers can be built upon or reinforced by personalising care. The data suggested that treatment adherence can be heavily influenced by tailoring the combined exercise and nutrition interventions to individual participants.

### 4.1. Knowledge and Skills

Education and improving knowledge are essential as part of delivering nutrition interventions, as a lack of knowledge/skills were reported as barriers towards participation and behavioural change [23,24]. This also applied to exercise. The provision of a personalised educational resource booklet complements the knowledge and skills taught during the intervention. Participants also valued the expertise of the healthcare provider. Their trust and confidence towards their healthcare providers brought about optimism about effect of the nutrition recommendations. Many participants described receiving advice that was consistent to the program, (i.e., their general practitioner (GP) also encouraged them to engage in behaviours around nutrition and exercise). This has been previously reported where regular contact with GPs was highlighted as a facilitator towards exercise and protein-rich foods [25,26]. The present study therefore emphasised the importance of consistent advice between healthcare providers across health systems.

### 4.2. Environmental Context and Resources

The data suggest that it is essential to consider the participant’s existing health conditions that may limit the extent of adherence. For nutrition support to be effective, nutrition-related goals ideally need to be individualised by a trained dietitian, e.g., a protein set higher to adjust for increased breakdown during acute disease and lower targets for those with renal issues [27]. A combination of dietary issues and restrictions from multiple concurrent conditions present challenges towards nutritional adequacy. The data suggested that when prescribing diet recommendations and ONS, physical limitations to pre-frail and frail older adults should also be considered. Pre-frail and frail older adults may have disabilities limiting their mobility, and thus ability to prepare food. Conditions such as arthritis can affect grip and food preparation [28]. For example, there could be difficulties in performing basic kitchen-related tasks such as opening a cap [29]. A previous study also reported that frailty was described to be a physical limitation to preparing food: a lack of strength to cook [30]. Limited transport has also been cited previously as barrier towards healthy eating and disease self-management among older adults [31].

The perceived barrier of a lack of energy and time when it comes to exercise have both been reported many times in the previous literature [32,33]. This would suggest that healthcare providers need to address these issues by tailoring prescribed exercises, keeping in mind the length of the session, the time set for set-up, and transportation. A review suggested that the enjoyment of physical activity relies on individual preferences for location (indoors or outdoors) [34]. Participants in this cohort were asked to walk three times a week in the program they participated in. However, wet/cold weather was cited as a barrier to exercise in this study and could have impacted adherence to the walking component. Chan and Ryan [35] found a negative correlation between rainfall and physical activity. Considering that fact that the exercises (other than walking) recommended were indoors, the effects of wet weather on exercise may be affected more by environmental factors other than logistical reasons.

### 4.3. Social Influences

Loneliness impacts dietary adherence and choice greatly. The perceived lack of social support or social opposition have been reported across other populations as well [36]. Older adults with partners/family members described social support as a significant enabler to dietary adherence to the program. It has been previously reported that older adults living with their partners tended to have more variety and larger meal sizes [37]. Studies have also shown shared meals to be effective in improving energy intake, albeit demonstrated in institutionalised older adults [38]. Social support is a frailty domain and is assessed within the EFS. The finding of loneliness and peer pressure correspond to another study citing the lack of social support and social opposition to be barriers towards dietary and exercise adherence [39]. Social frailty has recently garnered increasing interest, with some studies defining it as insufficient or as having no participation in social networks with a perception of absence of contacts/support [40,41].

### 4.4. Emotion

A range of emotions (fear, lack of enjoyment and appetite, and depressive mood) that negatively impact dietary adherence were described in this study. The lack of appetite and enjoyment could be linked to changes across physiological systems. Reduced hunger and early satiety are common issues raised by older adults; ageing is related to many physiological changes that favour reduced intake [42]. There are ways to respond to that barrier using a patient-centred approach [43]. Pre-prepared meals in the forms of ready-to-eat chilled or frozen meals can keep nutritional intake adequate when there is a lack motivation to cook. The lack of enjoyment presents opportunities for healthcare providers to get creative. Again, individualised support, as opposed to prescribing more ONS, would help the participant navigate around this issue (i.e., improving the flavours of food with stronger herbs and spices). The simple process of matching food/supplement prescriptions with preferences is often overlooked. Emotions identified as barriers to exercises were stress from dealing with pain, depression and anxiety, and fear of pending medical diagnosis. Depressive symptoms and perceived poor health were described as a barrier to physical activity and exercise [44,45]. The range of emotional factors affecting adherence could be alleviated by the inclusion of psychological interventions. Future programs can consider including components of psychological interventions as needed [46]. Social support was also reported to be negatively associated with depressive mood [47], highlighting the importance of the social aspect of any pre-frailty and frailty when it comes to overcoming barriers to program adherence.

### 4.5. Strength and Limitations

This study captured a wide range of views of participants from a range of ages, nutritional status, and degrees of frailty, including pre-frailty. The interview guide protocol was developed with reference to the literature of combined exercise and nutrition interventions and a range of clinical expertise of the investigators representing different fields within pre-frailty and frailty care, and not based on the TDF. Using this method, the questions and responses were direct and thus more focused to the program, rather than restricted to questions specific to each of the theoretical domains. Notwithstanding that, all codes in this study could be matched to a domain within the TDF, further highlighting the framework’s comprehensiveness.

A limitation of this study was that the researcher that conducted the interviews also delivered the program’s intervention. Social desirability bias may have occurred where participants exaggerated positive aspects of the program or could not report all personal barriers. Measures put in place such as briefing the participant about the role of the interviewer as a researcher, anonymity using pseudonyms, and the non-involvement of formal care within the Local Health Network might have lessened said bias. A possible benefit of conducting the interview using with the researcher who also delivered the intervention might be his status as an “insider” and perceived as an “advocate” for the participant. This may allow for more thorough engagement in issues, encourage disclosure, and elicit a more “private” account and richer data [48,49]. It may also be possible that participants would be more inclined to share more sensitive issues or unpopular opinions. A study suggested that shared knowledge and interest between the interviewer and participants may boost the interviewer’s credibility [50]. While the use of an independent person unrelated to the program can reduce bias, their status as an “outsider” may also elicit reticence. There were also additional checks for accuracy between the transcription and recording by another member of the research team.

## 5. Conclusions

Healthcare providers and policy makers planning interventions to alleviate pre-frailty and frailty should be aware of the multitude of barriers and enablers such as environmental, psychosocial influences affecting the adherence to a self-managed combined exercise-and-nutrition program in this population. The individualisation of such nutrition–exercise services could help overcome said barriers and possibly improve their adherence. While self-managed programs such as this can be patient-led, the availability of human resources in the context of formal support from health professionals and informal ones from their social circle will still play a pivotal part in their success. Nevertheless, potential self-managed programs targeting pre-frail and frail older adults would require a multifaceted approach while tackling these barriers and putting in efforts to expand enablers that this study found.

## Figures and Tables

**Table 1:** Demographic and health characteristics of study participants (*n* = 11).

Characteristics	Interview Cohort (*n* = 11)
Age, years, mean ± SD	80.4 ± 6.3
Female, *n* (%)	7 (64%)
BMI ^a^, kg/m^2^, mean ± SD	26.1 ± 6.2
MMSE score ^b^, mean ± SD	28.3 ± 1.4
Charlson Comorbidity Index, mean ± SD	4.6 ± 1.7
Tertiary education level	3 (27%)
Living alone	6 (55%)
Mean Edmonton Frail Scale	8.9 ± 1.9
Pre-frail	2 (18%)
Frail	9 (82%)
PG-SGA grade—Malnourished, *n* (%)	6 (55%)
Scored PG-SGA, mean ± SD	7.9 ± 3.8
Short Physical Performance Battery, mean ± SD	2.6 ± 1.8
Geriatric Depression Scale, mean ± SD	5.6 ± 3.6
EQ-5D-5L Utility Index ^c^, mean ± SD	0.37 ± 0.37
EQ-5D VAS ^d^, mean ± SD	61.6 ± 23.9

Data expressed as mean ± SD for continuous variables; absolute numbers (percentage) for categorical variables. ^a^ Body Mass Index. ^b^ Mini Mental State Examinations. ^c^ Quality of life measured with the EQ-5D-5L utility index: has scores between 0 and 1 with higher scores indicating better health-related quality of life. ^d^ Quality of life measured with the EQ-5D visual analogue: has scores between 0 and 100 with higher scores indicating better health-related quality of life.

**Table 2:** Sample quotes of experience within the INDEPENDENCE program by codes.

TDF Domain	Codes	Illustrative Quotations
Knowledge	Increased awareness about lifestyle behaviours	“*I’ve not thought so much about diet, so it made me think about diet a little bit more on what I was eating in in terms of exercise, because I was fairly active before the exercise component.*” “*I think the benefit the benefit is mainly being that I become more diet conscious.*”
Social identity	Participant of a program	*“I just think it was really helpful that I that [name of therapist] invited me to connect into this program, it’s been really helpful.”*
Beliefs about capabilities	Perceived lack of ability to participate	“*Well, all my life I was born with a muscle weakness yes. And all my life was a struggle.*” “*I’m so sorry to say and I just can’t do it because I enjoy his visits and his advice, but I can’t do it.*”
Optimism	Optimism about effects of program	*“Getting used to eating and have regular meals. That was the most important thing for me and be able to walk again and to move again.”*
Reinforcement	Reward of getting back to pre-hospitalised activities	*“I did get back to you know, sorting out some stamps and picked up my ukulele and trying to get back in things.”*
Environmental context/resource	Disruptions to program by readmissions	*“Yeah, yeah, in admissions into hospital ‘cause I injured my leg then I’ve had that funny thing that grew on the top of my foot and so it interrupted it all. It wasn’t a continuous flow.”*
	Lack of time for program	*“I didn’t expect that when I came out of hospital, I thought I was going to have time.”*
	Positive affect for home visits	*“I think the home visits have been very helpful.”*
	Flexibility of program	*“Yeah, I like that arrangement. I looked in my on the weekend. I looked in my diary for today and I thought, oh yes, three o’clock OK, I just like that planning ahead thing that.”*
Social influence	Individualised support	“*This was more personal. You know the help that you I get at each time that [name of home therapist] came out. And then I am improved as I went on*.” “*But [name of therapist] is beautiful, I love to participate in the program. He is a beautiful person, and he brings it in. Acceptable they and I have really appreciated him.”*
Behavioural regulation	Habit forming	*“I’m quite happy to do them. And try to do them at least before 2 meals.”*

**Table 3:** Sample quotes of barriers to the nutrition and exercise components of the INDEPENDENCE program by codes.

TDF Domain	Codes	Illustrative Quotations
Beliefs about capabilities	Lack of coordination and balance (E)	*“I can’t stand on my feet very well”*
Intentions	Lack of motivation to cook (N)	*“Only laziness”* *“Or I don’t feel like cooking”*
	Lack of internal motivation (E)	*“I didn’t have the motivation to do it”* *“I don’t think I have much motivation”*
Memory, attention, and decision processes	Forgetfulness (E)	*“It just doesn’t come up. I don’t think about it. A lot of the times that I should be doing it.”*
Environmental context/resource	Dietary restrictions from multimorbidity (N)	*“I’ve got to stick virtually the three different diets don’t I low sodium gout diet and potassium diet, don’t I, you know?”*
	Side effect from medications/treatments (N)	*“When they give me the first injection, I was sick in the stomach. I couldn’t eat. I had to force myself to eat”*
	Limited transport (N)	*“If I forget something or if I feel like something I can’t get it because there’s no way really. Well, there’s no transport around here”*
	Physical limitation to prepare food (N)	*“Yeah, the rheumatoid yeah. I’m only you know if I go to lift the pan or a sauce pan up, I find it very hard.”*
	Cold/wet weather (E)	*“Too cold and wet….and when it’s wet, I don’t go out for a sweat”* *“Right now, cold weather”*
	Comorbidities and injuries (E)	*“Itch… it’s constant. I didn’t get any sleep. So, you know it was very hard you see. It consumed my life.”*
	Lack of sleep (E)	*“You know and lack of sleep.”*
	Lack of energy (E)	*“No, yeah, well I was tired. So tired.”*
	Lack of time (E)	*“The simple answer is time… you know, routines even within the house. And the day just never seems long enough”*
Social influence	Loneliness (N)	*“Eating alone, I think I’ve told you haven’t heard that. My husband had restaurants and yes, and I’m, you know, always get stuff cooked for me and then you know I go into the restaurant and if he’s got people that are coming in, I sit down and eat with them. And yeah, yeah so”*
	Peer pressure (N)	*“The only thing that interferes with me doing it is it I go out and put people like people asking me out. And give me a meal I feel obliged to eat what they put in front of me”*
	Prioritising social activities over exercise (E)	*“If I get an invitation to go out somewhere with somebody, I’ll drop the exercise and go out.”*
Emotion	Lack of enjoyment (N)	*“I put pressure on myself to eat something and I used to be a good eater. Uh, food used to be celebration for me and now food is a punishment”* *‘I still have trouble tasting. I don’t get the nice time able to drink tea now.”*
	Fear of insufficient funds (N)	*“I was afraid that my money wouldn’t go as far as I needed it to go, but it was just unreasonable fear.”*
	Lack of appetite (N)	*“Oh, especially now sometimes I just don’t feel hungry at all. But I’m forcing myself to eat.”*
	Depressive/anxious mood (N) (E)	*“Now I hate getting out. I just like staying in my bed. I think the earlier I get up, the longer the day is.”* “*All depression and anxiety*”
	Stress from dealing with physical ailments (E)	“*Pain, yeah. Just the pain that it caused me and then. I have to do it in the kitchen because I can hold on to the bench and then I can’t get back, but there’s only so much pain here*”
	Fear from pending diagnosis (E)	“*I was waiting to get the results. From this these tests. No, no waiting for them to give me the green light so that I didn’t overdo it*”
Behavioural regulation	Not part of daily routine (E)	“*Yeah, I just never thought of it. Perhaps that day*.”

Note: (E) indicates codes identified during the exercise component of the INDEPENDENCE program, while (N) indicates codes identified during the program’s nutrition component.

**Table 4:** Sample quotes about enablers to the nutrition and exercise components in the INDEPENDENCE program by codes.

TDF Domain	Codes	Illustrative Quotations
Knowledge	Improved awareness and knowledge of food and health (E)	*“what the program did was made me look at what we were preparing or what was on the plate. And making sure that we had a good balance of food but with a bias towards the proteins.”* *“Yeah, I’ll make sure I put some protein in it”*
	Increased importance of protein (N)	*“Yeah, I’ll make sure I put some protein in it”*
	Increased awareness of importance to not stay sedentary (E)	“*I’m more aware that I’m sitting. I need to move. Yeah, yeah. So I am more aware of that, yes*?”
Skills	Consistent dietary advice from healthcare staff across services (N)	*“I think it was seamless from the hospital and you and my GP. And before I think I was using water. So, to put the sustagen, mix it with water.”* *“Right, yeah, well understand about the portions bit better from (home visit therapist).”*
	Portioning of food (N)	*“Right, yeah, well understand about the portions bit better from (home visit therapist).”*
	Less reliant on gait aids (E)	“*at times I don’t even use the Walker or, you know I can open if I’ve got something to grab on that I can. Do that*” “*I don’t find it difficult at all*.” “*You’ve explained yourself so well, and I haven’t felt. The need to call you*”
	Level of difficulty of home exercises were manageable (E)	“*I don’t find it difficult at all*.”
	Learnt skill from healthcare provider (E)	“*You’ve explained yourself so well, and I haven’t felt. The need to call you*”
Social identity	Caregiver to others (N)	*“Yeah, it’s. She has dementia. So, it’s where she needs me badly”*
	Participant of a program (E)	*“I thought, you know, I’ve started it. I’ve got to finish it.”*
	Independent person/parent (E)	*“As I say. Some people expect their child to be there every five minutes, and that’s just not the way that we are... I just think sometimes that some parents think expect too much of their children because they’ve got their own lives. They’ve got their own family to look after. So there. You really should. Be stronger and more independent.”*
	Worthy patient (E)	*“They said that they see me see me as his old. He is a patient worth working on. Otherwise, once you get over 80, they’re not interested”*
Beliefs about capabilities	Perceived competence from previous program (E)	*“I mean just doing them more often and you know I’ve always done a lot of walking anyway. You know but haven’t necessarily done all the other things.”*
Optimism	Trust and confidence in healthcare provider (N)	*“Yeah, and I respected the fact, I suppose that [therapist] done that. [therapist] have done the hard yards and got qualification in it”*
	Confidence in prescribed exercises (E)	*“I think the answer is the exercises when they’re given by an external organisation rather than me just thinking them up myself. You know, they have been developed for a reason.”*
	Optimism of increasing physical capabilities (E)	*“This though, because I think I might get better”* *“I’ve liked doing the exercise to getting out and seeing what I can do.”*
Beliefs about consequences	Perception that nutrition will improve weight/strength (N)	*“Realising that I had to work on the dietary program, but I did find having lost 12 kilos in weight between going to hospital and coming out the [hospital].”*
	Perception that nutrition can improve blood glucose (N)	*“That’s an obvious improvement. Which is helping with the BGL, because that’s coming down.”*
	Perception that exercise will maintain or improve strength/health (E)	*“I know if I don’t keep the exercise up that my strengths not going to come back, you know. So, I’ve tried to do them when I can, yeah”* *“And I knew it was going to build up stamina and give me strength again. So there was a big incentive”*
	Perception that exercise helped maintain independence (E)	*“Well, I like to keep fit to a certain extent. I’m on my own so I have to do things for myself. Yes, that’s how I try to keep fit to enable me to do them”*
	Perception that exercise will improve appetite (E)	*“I’m underweight and I feel that it may improve my appetite with the exercises”*
	Accountability to health-support workers (E)	*“I think when you realise that you have some supervision, you have some external help. These sorts of things are motivators as well that you know you are wanting to do it for yourself, but you’re wanting to do it for the tutor so that they can see the benefit of their work and their recommendations”*
	Fall prevention (E)	*“Actually, we’re keeping the lower body very strong, which enables us not to fall over, which is the whole idea.”*
Reinforcement	Exercise-induced appetite (N)	*“I felt with the exercise it did give me a little more appetite than I had before”*
	Mental benefits (N)	*“Because I’m I feel better.”*
	Physical/health benefits (E)	*“Yeah and getting up out of the chair. A great deal”* *“These simple exercises done around the chair and their added weight in nature. Uhm, they have more effect on the weight control and the sugar control than what the other exercises I do.”*
Intentions	Intrinsic motivation (E)	*“I say to myself, I’ve got to do them, and I do them.”* *“My own will power”* *“Sometimes if I’m busy or going to be busy. I’ll just go along a walk up on the block that went along halls. You know?”*
	Constant decision to stay active (E)	*“Sometimes if I’m busy or going to be busy. I’ll just go along a walk up on the block that went along halls. You know?”*
Goals	Weight gain (N)	*“I’ve got the scales in the bathroom. I weigh myself every morning when I get out of the chair and I’m just hoping to see that. It’s really not. Yeah. Get up a little bit more than it has”*
	To reclaim life prior to hospitalisation (E)	*“You, well, you know I wanted to, you know, resume my life and start instead of watching the church service on the tablet that I could go back to church as well, you know”*
Memory, attention, and decision processes	No reliance on memory for exercises (E)	*“The fact that all the exercises are illustrated so that you can’t make a mistake because you can see it.”*
Environmental context/resource	Government funding (N)	*“No, it [government funding support] helps me because something go out shopping, buy anything I want, you know”*
	Meal-delivery service (N)	*“Well, you know I was on light and easy (pre-prepared meal program)”*
	Education resource booklet (E) (N)	*“Yeah, like it’s you know I try to refer to this book”* *“Well, I do the exercises in the book, but perhaps without I wouldn’t have done them so often”*
	Presence of outdoor facility (E)	*“There’s an open-air gym down the road. There we go down there”*
Social influences	Support from family (E) (N)	*“[Daughter] suggested that she get high protein milk for the coffee because she said that’ll take care of two proteins straight away. So, then I only had to worry about 5.”*
	Company of a spouse (N)	*“I’m very lucky to still have my partner so someone I can talk to and eat with and prepare food with is very beneficial”* *“Must my wife and myself. And she was an in great Encourager all the time but she never really interfered with me”*
	Support from peers (N)	*“Oh, people encouraging me and saying gosh, you’re looking good”*
	Support from healthcare providers (E)	*“The very fact that [therapist] come here and we do what we do and then [therapist] ring up and we talk about what we talk about. It’s just that continual connection, yeah?.”*
Emotions	Increased enjoyment in food/supplements (N)	*“Well, it’s it seems to be taste here. It tastes smoother, Yes, yeah I do. I do like the protein drinks I really do like them”*
Behavioural regulation	Fitting exercises into daily routine (E)	*“Trying to get the exercises in, especially when I get about to go out”* *“Yeah, I should get up in the morning after breakfast. Do my exercise, then get on with the rest of the day.”*

## Data Availability

Data are contained within the article.

## Supplementary Materials

The following supporting information can be downloaded at: https://www.mdpi.com/article/10.3390/healthcare12060678/s1, Supplementary File S1: COREQ statement/checklist [15]. Supplementary File S2: Full interview guide.
