# Supplementary material for: Barriers and Enablers to a Hospital-to-Home, Combined Exercise and Nutrition, Self-Managed Program for Pre-Frail and Frail Hospitalised Older Adults
Source: Healthcare (Basel). 2024 Mar 18;12(6):678. doi: 10.3390/healthcare12060678 (PMC10970091; doi:10.3390/healthcare12060678)
Supplement: Supplementary file 1 [file healthcare-12-00678-s001.zip › Online Supplementary File S1.pdf]

**Supplementary File S1**

**COREQ (COnsolidated criteria for REporting Qualitative research) 32 item Checklist**

Tong A, Sainsbury P, Craig J. Consolidated criteria for reporting qualitative research (COREQ): a 32-item checklist for interviews and focus groups. International Journal for Quality in Health Care. 2007. Volume 19, Number 6: pp. 349 – 357

| Item Number | Domain | Comment | Reported on page number or not applicable (N/A) |
|---|---|---|---|
| | ***Domain 1: Research team and reflexivity*** | | |
| | Personal characteristics | | |
| 1 | Interviewer/facilitator | Research Dietitian (Dr Chad Han, C.Y.H.) conducted the interviews | 2-3 |
| 2 | Credentials | Five/Six research team members have prior experience of conducting qualitative health research – C.Y.H., G.M., A.Y., Y.S., C.B., M.M. and J.D. completed formal certification course in qualitative analysis course. | 2-3 |
| 3 | Occupation | C.Y.H. – accredited practicing dietitian and PhD in nutrition and dietetics focusing on frailty, G.M., PhD using qualitative methods at time of analysis, A.Y., accredited practicing dietitian, senior lecturer in nutrition and dietetics, Y.S., Senior consultant and A/Professor in Medicine; C.B., Physiotherapist and Senior Lecturer in Physiotherapy M.M. advanced accredited practicing dietitian and Professor of nutrition and dietetics; J.D., graduate of Bachelors of Linguistic and Psychology | 2-3 |
| 4 | Gender | Two male-identifying and four female identifying researchers (interviewer, male-identifying) | 2-3 |
| 5 | Experience and training | Six/seven research team members have prior experience of conducting qualitative health research – C.Y.H., G.M., A.Y., Y.S., C.B., M.M. and J.D. completed formal certification course in qualitative analysis course. | 2-3 |
| | Relationship with participants | | |
| 6 | Relationship established | The interviewer had no relationships with participants pertaining to the healthcare services prior to the study, while two of research team members (C.B.; Y.S.) had varying relationships with the health community at large. (C.Y.H.) directly involved with participants was competent in clinical management of pre-frail/frail older adults within their professional scope. This fostered a trusting relationship between the experiment facilitators and patients in order to produce accurate findings and preserve the quality of the research. It was felt the interviewer should be seen as an unbiased enquirer rather than a person with a particular stance or opinion about the topic. | 2-3 |
| 7 | Participant knowledge of the interviewer | The interviewer introduced himself to participants as a researcher and cannot and will not affect their access to healthcare and health systems, as well as describing the research team, its funding, the purpose of the project and answering any questions participants may have had about the project and those involved in it. | 3 |

| | | | |
|---|---|---|---|
| 8 | Interviewer characteristics | The interviewer was an Australian trained accredited pracitising dietitian, aware of the medical system in Australia and had read the literature on care pathways prior to beginning the project. | |
| | ***Domain 2: Study design*** | | |
| | ***Theoretical framework*** | | |
| 9 | Methodological orientation and theory | Deductive thematic analysis using the Consolidated Framework for Implementation Research | 3-4 |
| | Participant selection | | |
| 10 | Sampling | Purposive sampling was used | 3 |
| 11 | Method of approach | Participants were approached took part in a hospital-to-home, self-managed combined exercise and nutrition intervention program between September 2020 to June 2021. | 3 |
| 12 | Sample size | 11 | 4 |
| 13 | Non-participation | Four out of the 16 total participants recruited in the study did not participate in this study – three were deceased and one declined. | 3 |
| | Setting | | |
| 14 | Setting of data collection | All interviews were conducted in homes of the participant to ensure their privacy, and to allow them to freely express their personal views. Participants were also interviewed privately, in the absence of other family members, where pseudonyms were used to ensure anonymity the recording. | 3 |
| 15 | Presence of non-participant | None | N/A |
| 16 | Description of sample | Fully presented in results section | 4; table 1 |
| | Data collection | | |
| 17 | Interview guide | The interview guide was semi-structed and listed a number of key questions and potential prompts. Interviews generally followed a similar approach although questions themselves varied depending on the participant. | Online supplementary materials 2 |
| 18 | Repeat interviews | No | N/A |
| 19 | Audio/visual recording | All interviews were audio-recorded and later transcribed. | 3 |
| 20 | Field notes | None taken | N/A |
| 21 | Duration | Interview times ranged from 15 to 60 minutes | 4 |
| 22 | Data saturation | Not applicable as a deductive thematic analysis | N/A |
| 23 | Transcripts returned | No | N/A |
| | ***Domain 3: analysis and findings*** | | |
| | ***Data analysis*** | | |
| 24 | Number of data coders | The interviewer (C.Y.H.) transcribed the interviews, and the transcriptions were cross-checked by another member of the research team (J.D.). C.Y.H. was responsible for data analysis, and the coded transcripts were checked by two other members of the research team (C.B., A.Y.). | 3-4 |
| 25 | Description of the coding tree | Codes were tagged and description within the software except for where they were self-explanatory. | Tables 2, 3, 4 |
| 26 | Derivation of themes | The themes were those used in the Theoretical Domains Framework. | Tables 2, 3, 4 |
| 27 | Software | Microsoft word was used. | 3 |
| 28 | Participant checking | No | N/A |
| | Reporting | | |
| 29 | Quotations presented | Yes, quotations are presented and identified in a manner protecting participants confidentiality | Tables 2, 3, 4 |

| | | | |
|---|---|---|---|
| 30 | Data and findings consistent | Consistency between the data and the findings exists | 4-16 |
| 31 | Clarity of major themes | Yes, major themes are clearly identified and matched to the domains within the theoretics domains framework (TDF) | Tables 2, 3, 4 |
| 32 | Clarity of minor themes | Yes, minor themes are defined and matched to the domains within the theoretics domains framework (TDF) | Tables 2, 3, 4 |
