# Supplementary material for: Barriers and Enablers to a Hospital-to-Home, Combined Exercise and Nutrition, Self-Managed Program for Pre-Frail and Frail Hospitalised Older Adults
Source: Healthcare (Basel). 2024 Mar 18;12(6):678. doi: 10.3390/healthcare12060678 (PMC10970091; doi:10.3390/healthcare12060678)
Supplement: Supplementary file 1 [file healthcare-12-00678-s001.zip › Online Supplementary File S2.pdf]

Online Supplementary File S2

Instructions

Good morning (afternoon). My name is ________. Thank you for participating. In this interview, I will ask you about your experiences and opinions as a participant in the hospital to home support program which you took part in the past 3 months. The purpose is to get your perceptions of your experiences and opinions around this new service.

I must highlight that there are no right or wrong, desirable or undesirable answers. I would like you to feel comfortable with saying what you really think and how you really feel.

Audio recorder instructions

If it is okay with you, I will be recording our conversation. The purpose of this is so that I can get all the details but at the same time be able to carry on an attentive conversation with you. I assure you that all your comments will remain confidential. I will be compiling a report which will contain all participants' comments without any reference to individuals.

Part 1. Introduction – Getting both parties comfortable with the conversation *(keep to 5 minutes)*

- How is a typical day for you like since you were home from the last hospital stay?
- How long has it been since you started participating in this hospital to home support program?
- In your opinion, what part of the program has been working well for you?
- What has not been working well for you? What could be different?

Part 2. Open conversation – Seeking stories about their life, work or event about which you are trying to find out?

I will now ask you about your experience on changing your diet

- What are some of the experiences or help that enabled you to keep to the dietary advice and changes within this hospital to home program? (*prompts: what stopped you from achieving your diet goals?)*
- Have you had any experiences that prevented you from making the changes to your diet? (*prompts: what stopped you from achieving your diet goals?)*

Thank you for sharing, I will now ask you about your experience on doing home exercises

- I will first ask you about your experience about doing home exercises
- What are some of the experiences or help that enabled you to keep to the exercises with your health care professional? (*prompts: what help you achieve your exercise goals?)*
- Have you had any experiences that prevented you from keeping to the exercises? (*prompts: what stopped you from achieving your exercise goals?)*

Part 3. Clean up – Ask specific questions that have not been addressed, become provocative, or ask standard questions for comparison

- What are some of the factors that helped you stick to the dietary changes and exercises in past 3 months? (prompts: *Like something that helped you eat better and exercising)*
- What are some difficulties that you face when trying to keep to the dietary changes and exercises in past 3 months? (prompts: *Like something that stopped you from eating better and exercising)*

Part 4. Wrap up – Ask if there is anything the respondent would like to add, anything they want you to know

We have come to the end of our questions. I would like to reassure you that all your responses are kept strictly confidential.

Now that you know what the interview was about, and you know what we've been talking about, is there anything else you would like to share that we did not talk about?

Is there anything else that you would like to highlight to me?

Prompts and probes

- *Can you explain the situation? What occurred?*
- *How did you respond to that?*
- *What did you do next?*
- *Who was involved?*
- *Was there anything else that you could have done?*
- *How often did it happen?*
- *Who was involved? How did they respond?*
- *How was it resolved?*
- *Was this situation unique? Repeated?*
- *How would you have liked events to have worked?*
- *What do you mean by ______? Can you help me to understand this ______?*
